# Supplementary material for: Investigation of Somatic Mutations in Human Brains Targeting Genes Associated With Parkinson's Disease
Source: Front Neurol. 2020 Oct 22;11:570424. doi: 10.3389/fneur.2020.570424 (PMC7642339; doi:10.3389/fneur.2020.570424)
Supplement: Supplementary file 1 [file Data_Sheet_1.pdf]

Table S1. Demographics of each individual included in the Haloplex HS study. Samples used: substantia nigra (SN), frontal cortex (F), cerebellum (C) and blood (B)

Code	Condition	Age	Gender	Region/Tissue			
				SN	F	C	B
1	MSA	68	Male	X			
2	MSA	55	Female	X	X	X	
3	MSA	72	Male	X	X	X	X
4	ILB	93	Female	X			
5	PD	70	Male	X	X	X	
6	PD	73	Male	X	X	X	
7	PD	71	Male	X	X	X	
8	PD	64	Male	X			
9	PD	74	Male	X	X	X	
10	PD	61	tbc	X			
11	PD	65	Male	X			
12	PD	78	Male	X			
13	PD	79	Male	X			
14	PD	84	Female	X			
15	PD	72	Male	X	X	X	
16	PD	61	Male	X			
17	PD	78	Male	X	X		
18	PD	tbc	Male	X	X	X	
19	PD	55	Male	X			
20	PD	81	Male	X			
21	PD	63	Male	X			
22	PD	71	Male	X			
23	PD	72	Male	X			
24	PD	79	Female	X			
25	PD	85	Male	X			
26	PD	73	Male	X			
27	PD	80	Female	X	X	X	X
28	PD	64	Female	X			
29	PD	62	Male	X			
30	PD	88	Male	X	X	X	X
31	Control	94	Female	X			
32	Control	82	Male	X	X	X	
33	Control	92	Female	X			
34	Control	104	Female	X	X		
35	Control	76	Male	X			
36	Control	86	Female	X			
37	Control	82	Female	X			
38	Control	69	Male	X			
39	Control	89	Male	X			
40	Control	87	Female	X			
41	Control	79	Female	X			
42	Control	80	Female	X			
				42	13	11	3

Figure S1. Haloplex HS bioinformatic analysis. Data from HiSeq runs were converted from bcl to fastq files using CASAVA (version 1.8.2). Fastq files were analysed by FASTQC 0.11.5, to confirm no differences in quality between flow cell lanes. Customised trimming was performed by Trimmomatic 0.32. Fastq files from different lanes were merged into a single file for each sample using a mac terminal 2.7.3. Surecall 3.0 was used to de-duplicate, align and call variants in the sequenced samples. Variant calling was performed in Surecall using default values only with the adjustments for variant call quality (5) and minimum allele frequency (0.003). IGV 2.3.98 was used to assess candidate variants visually, passing only those meeting specific criteria. Variants were analysed by Polyphen-2 to predict their pathogenicity. As an additional analysis, LoFreq 2.1.2 and MuTect2 were used for a variant calling comparison against Surecall analysis in the artificial mosaic expected to carry variants at 1%.

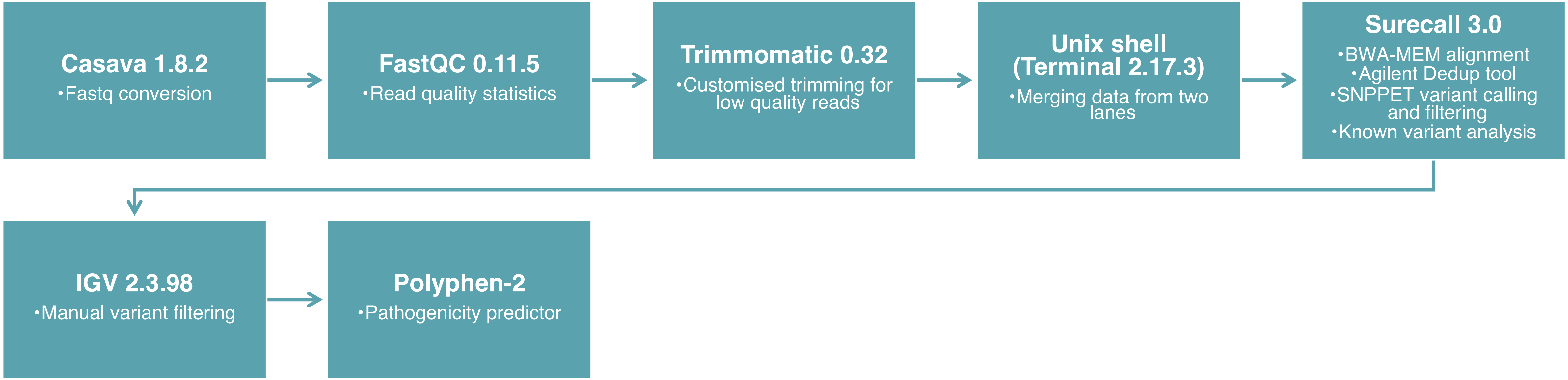

Table S2. Haloplex HS panel design and library preparation. The genes and probe coverage % with respect to the regions of interest are shown in the table. The targeted genes are associated to familial forms of PD, juvenile forms of Parkinsonism (such as *PLA2G6* and *ATP13A2*), and two genes associated to familial AD, frontotemporal dementia with Parkinsonism and reported previously to host somatic variants in AD brains (*APP* and *MAPT*; Frigerio, 2015). For sequencing library preparation, Genomic DNA quantification was performed using Qubit dsDNA BR assay and DNA quality was assessed with the Agilent NGS FFPE QC kit. To create 'artificial mosaics', we used two brain samples, one carrying SNPs in the region of interest and the other wild-type alleles at the same position. Samples were normalised at the same concentration, but mixed at different proportions to obtain the desired allele frequency (AF) for the SNPs of interest. We prepared Haloplex HS libraries according to the manufacturer, only adjusting for: (a) starting material (200 ng) to improve the detection of rare variants present in a few DNA molecules and (b) PCR cycles (22) to avoid overamplification of the sequencing libraries. Bioanalyzer HS DNA assay was used to assess the quality of sequencing libraries. Libraries were sequenced in a HiSeq 2500 platform, using the paired-end Rapid Run kit v2 (200 cycles).

Gene	Disease association	Coverage (%; relative to design targets)
SNCA	PD	91.16
GBA	PD	71.14
PARK7	PD	98.33
PINK1	PD	98.69
LRRK2	PD	94.85
PARK2	PD	94.09
PLA2G6	PD and dystonia-parkinsonism	96.83
APP	AD	95.5
VPS35	PD	93.56
FBX07	PD	99.94
ATP13A2	PD and Kufor-Rakeb syndrome	100
MAPT	FTD w/parkinsonism	96.91

Figure S2. Amplicon-sequencing bioinformatic analysis. Fastq files were processed using FASTQC 0.11.5 for quality control. Sequencing adapters were removed by Trimmomatic 0.36, using the trailing option to cut off bases at the read ends with quality lower than 30. BWA 0.7.15 was used for alignment and Picard 2.9.0 to sort and mark duplicates. Quality scores were adjusted with Base Quality Score Recalibrator from GATK 4.0.3 and variants were called by Mutect2. Each targeted region was individually analysed on IGV to assess if the sequencing reads showing the variant of interest were artefacts. A chi-square test with Yates correction was used to statistically compare the variant reads in samples and controls.

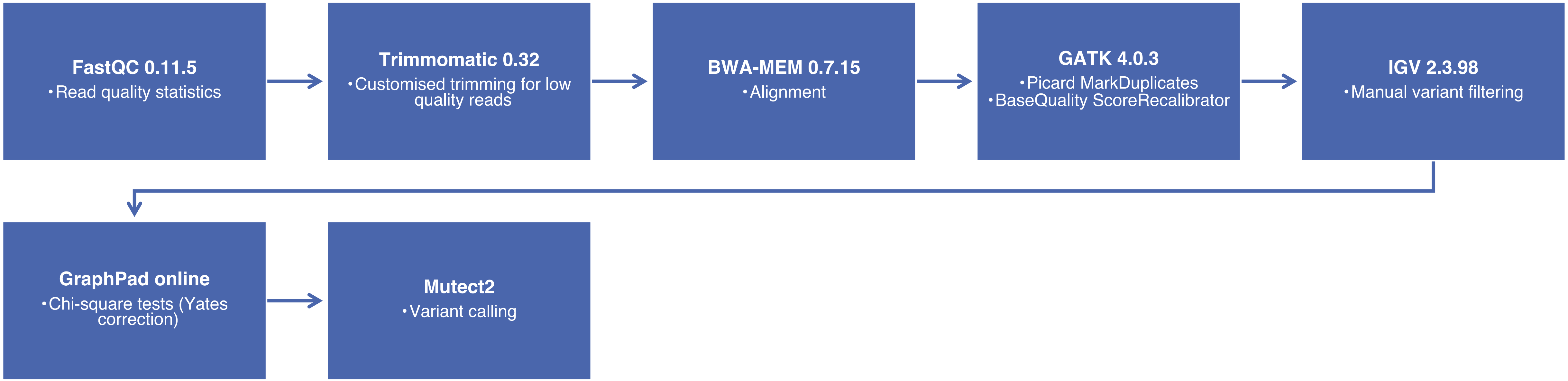

Table S3. ddPCR design, sample preparation and conditions. A shows the primers and probes targeting the variants in 34SN and B shows the primers and probes for the variant in 4SN. C shows the master mix concentrations for ddPCR assays. PCR reactions were run according to table D with annealing temperatures at 62.5ºC for mutation in 34SN sample and 65ºC for mutation in 4SN.

A

Name	Sequence
F_Primer	TCTAGGAGACCTGCCCA
F_Primer	GATGCTGGTGGCTTCTCTG
Ref_probe	CGCGACGTCGATGAGTCCTCCCC
Mut_probe	CGCGACGGCGATGAGTCCTCCCC

B

Name	Sequence
F_Primer	GACAGTAGTCAACGCTTCCC
F_Primer	CCAGACACTCCAACTCCCT
Ref_probe	TCTCGGGGGAGGAGTGTAGACAGTCGC
Mut_probe	TCTCGGGGGAGGAGAGTAGACAGTCGC

C

Reagents	Volume per reaction (ul)	Final concentration
2x ddPCR Supermix for Probes (no dUTP)	11	1x
20x primer forward	1.1	900 nM
20x primer reverse	1.1	900 nM
20x wild-type probe (HEX)	1.1	250 nM
20x mutant probe (FAM)	1.1	250 nM
DNA	1.1	5-40 ng/ul
Nuclease-free water	5.5	
Final reaction volume	22 ul	

D

Step	Temperature (ºC)	Time	Cycles
Enzyme activation	95	10 min	1
Denaturation	94	30 sec	40
Annealing/extension	Variable (see results)	1 min	
Elongation/enzyme deactivation	98	10 min	1
Hold	4		1

Figure S3. False positives estimated from the variant caller. A shows the PFP counts at different AF thresholds in each of the artificial mosaics. B shows the average fold increase (x) of Surecall variants when the detection limit was lowered from an AF percentage to the other (%→%). C shows a comparison of sensitivity registered for variants at 1% with Surecall and 2 additional variant callers.

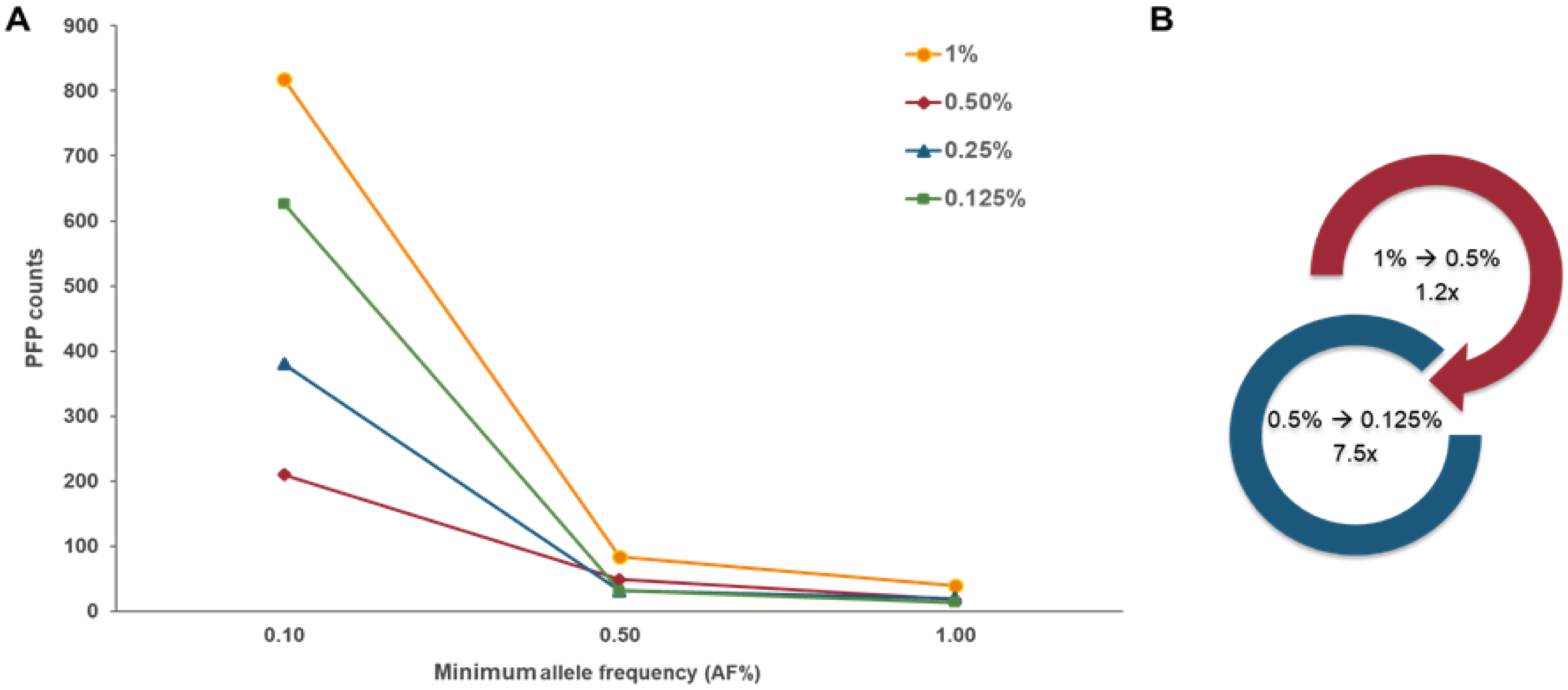

C

Variant caller	Sensitivity (%) for 1% AF
LoFreq	86
Surecall	92
Mutect2	30

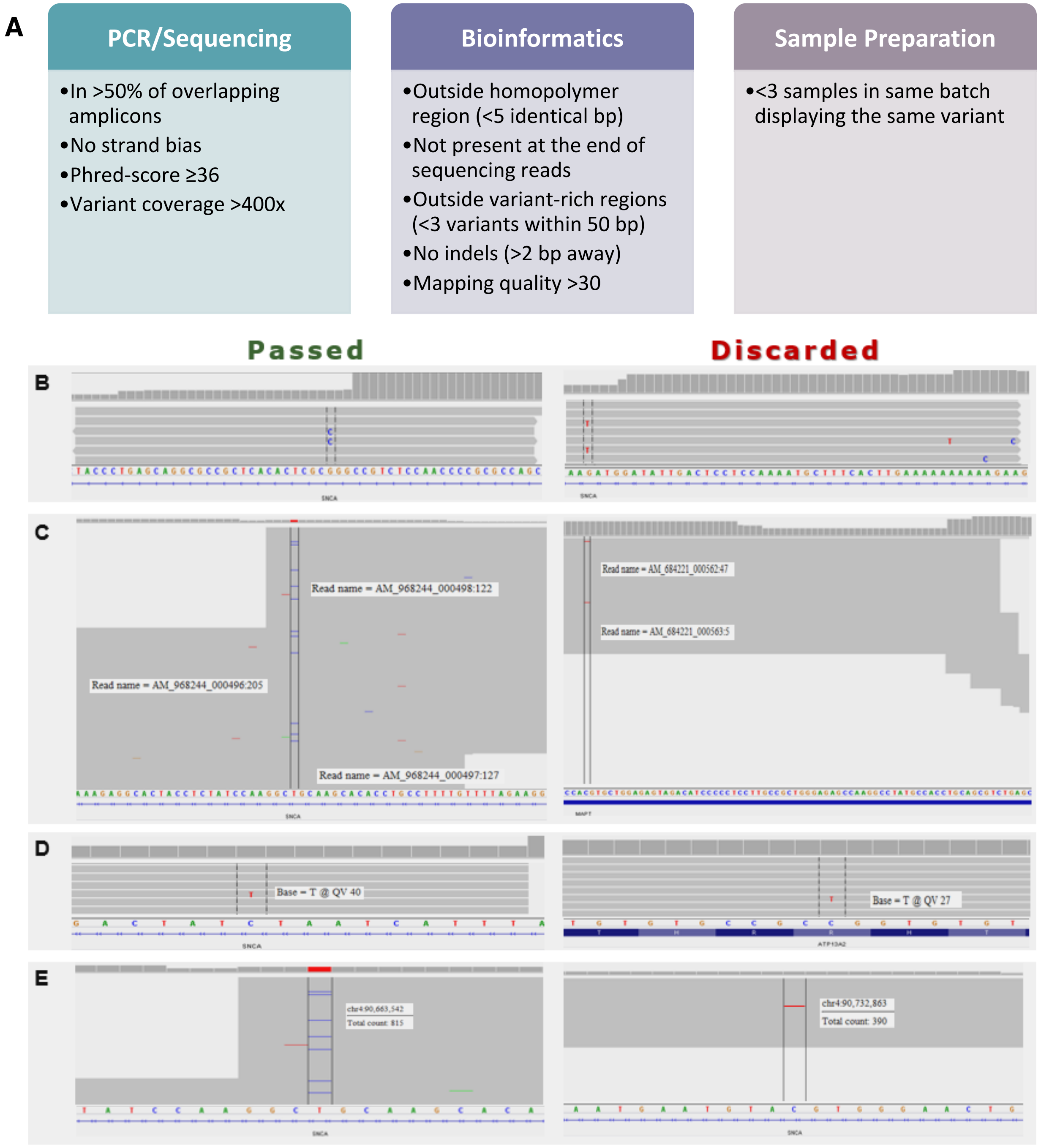

Figure S4. Filtering criteria used in IGV and examples of expected variants (passed) and false positives (discarded) after IGV analysis. A shows the criteria used in IGV to evaluate Surecall variants and discard artefacts from distinct error sources. B, C, D and E show examples of false positives which met all criteria, except but one, and were discarded from our analysis. For comparison, an example of a variant complying with the criteria is shown in each case. B shows a false positive with strand-bias, where only forward reads were carrying the false positive. C shows a false positive with no more than 50% of the overlapping amplicons were displaying the variant (AF= 0.57%). C shows a variant with phred-score < 35 (AF= 0.63%). D shows a variant with read-depth lower than 400x (AF= 1%).

Table S4. Ranking criteria used to prioritise variants for validation assays.

Ranking criteria	Score
Detected in PD/MSA cases	2
Allele frequency >1%	2
Allele frequency from 0.5-1%	1
>5 reads supporting alternate allele	1
Pathogenicity prediction by Polyphen-2	1
Substitutions not likely to be due to DNA damage (G>A, C>T, G>T, C>A)	1
Detected in Substantia nigra	1

A

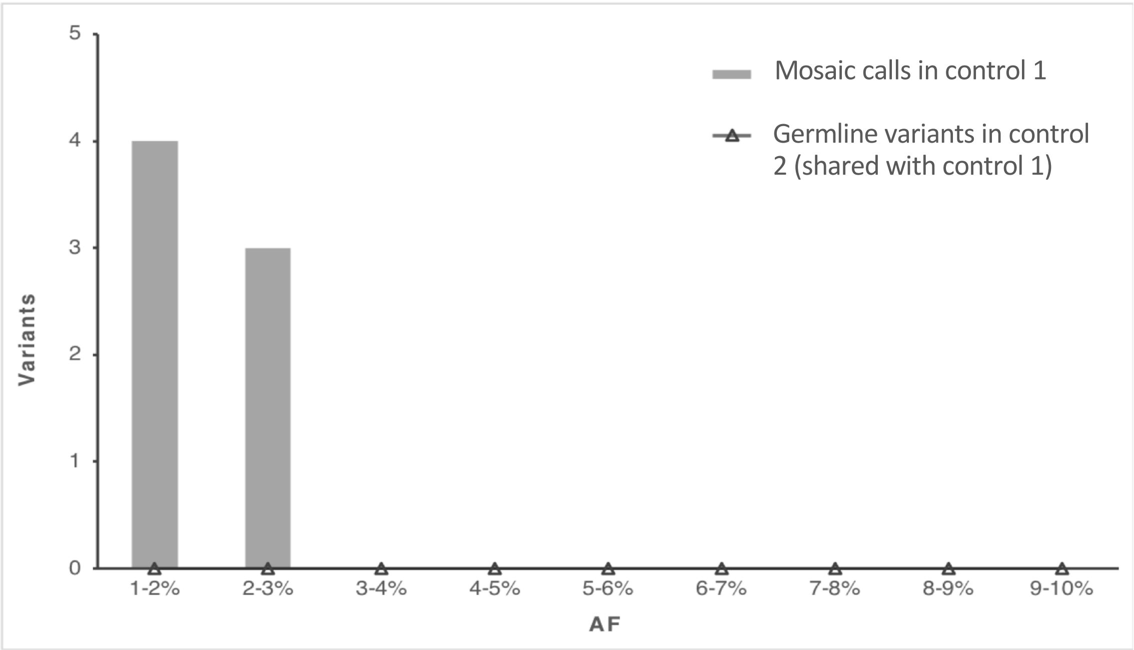

C

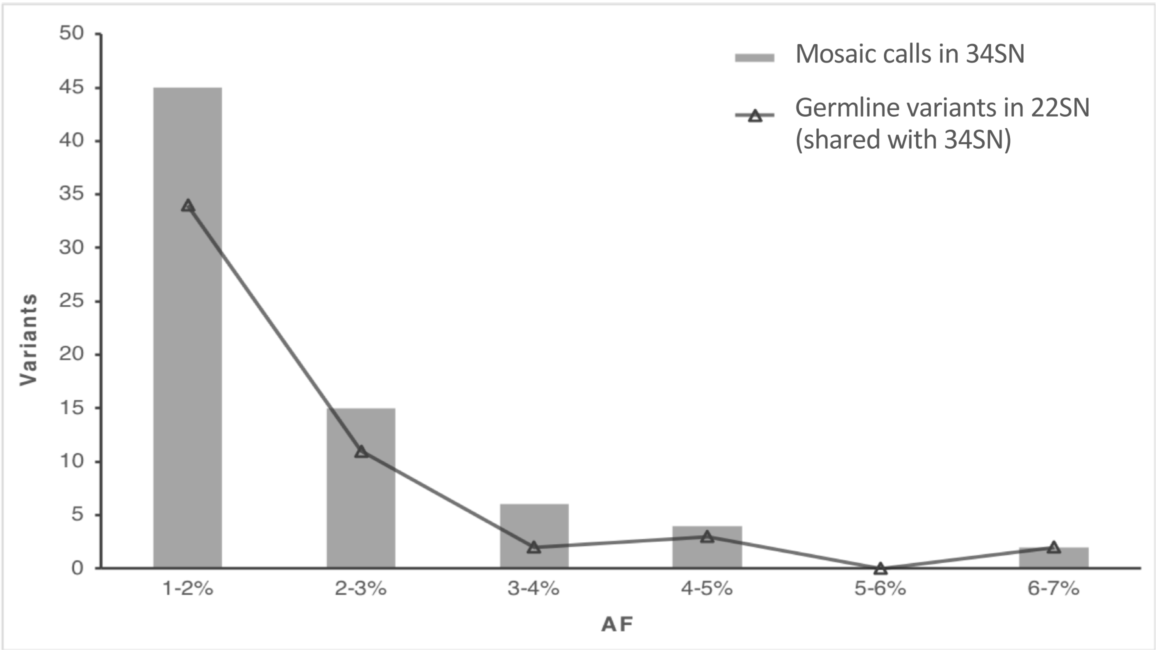

B

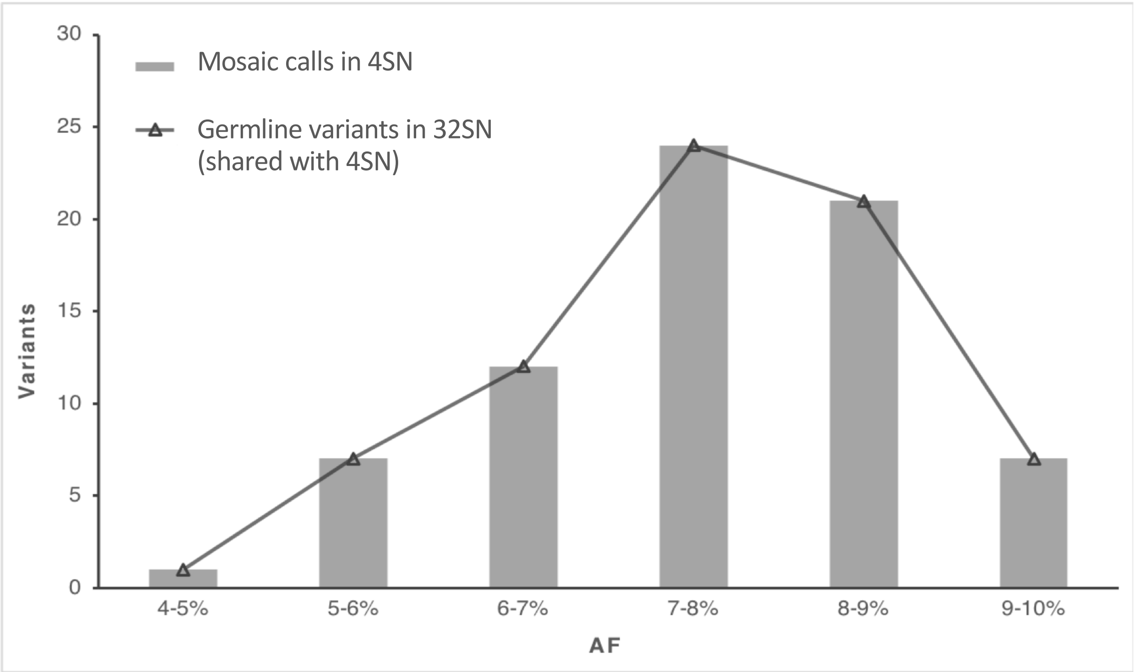

Figure S5. Contamination analysis by comparison of mosaic calls from likely contaminated samples against germline variants from the suspected contamination source. A shows control 1 vs. 2. The controls came from different brain banks, with DNA, library preparation and sequencing performed at different times. The two control samples show no similarity between the variants compared. B shows sample 34SN vs. sample 22SN and C shows sample 4SN vs. sample 32SN.

Supplementary Results from HiSeq runs

Hiseq2. Sequencing metrics were 93% of sequenced bases with quality values ≥30, average coverage of 2200x after de-duplication and 56.4% of duplicates. Estimated sensitivity for 1% variants was 92% and for 0.5% variants was 84%.

Hiseq3. Sequencing metrics were 94% of the bases with quality scores >30. Samples showed 2940x average depth after de-duplication and 49% of duplicates. The estimated sensitivity for 1% variants was 97% and for 0.5% variants the sensitivity was 90%. The sensitivity values were higher than previous HiSeq2 run, therefore the values were averaged and reported in the methods section
